# Long non-coding RNA SPRY4-IT1 promotes development of hepatic cellular carcinoma by interacting with ERRα and predicts poor prognosis

**DOI:** 10.1038/s41598-017-16781-9

**Published:** 2017-12-07

**Authors:** Guifang Yu, Jieheng Lin, Chengcheng Liu, Kailian Hou, Min Liang, Boyun Shi

**Affiliations:** The Fifth Affiliated Hospital of Guangzhou Medical University Guangzhou, Guangdong, China

## Abstract

Hepatocellular carcinoma (HCC) has become one of the most common leading causes of cancer-related deaths worldwide. This study investigates the role of lncRNA, SPRY4-IT1 in the development of HCC. Quantitative real-time PCR (qRT-PCR) was performed and the results showed that SPRY4-IT1 expression was up-regulated in HCC tissues and high expression of SPRY4-IT1 was associated with poor 5-year overall survival in the HCC patient cohort. Clinicopathological analysis showed that the expression of SPRY4-IT1 was significantly correlated with TNM stage in HCC patients. *In vitro* CCK-8 assay, colony formation assay, cell invasion and migration assays demonstrated that knock-down of SPRY4-IT1 suppressed cell proliferation, colony formation, cell invasion and migration in HCC cells. Flow cytometric analysis showed that knock-down of SPRY4-IT1 induced cell cycle arrest at G_0_/G_1_ phase and induced apoptosis. In addition, knock-down of SPRY4-IT1 also suppressed the mRNA and protein expression of estrogen-related receptor α (ERRα). Similarly, knock-down of ERRα inhibited cell proliferation, colony formation, cell invasion and migration in HCC cells. More importantly, ERRα overexpression antagonized the effects of SPRY4-IT1 knock-down on cell proliferation, colony formation, cell invasion and migration in HCC cells. Taken together, our data highlights the pivotal role of SPRY4-IT1 in the tumorigenesis of HCC.

## Introduction

Hepatocellular carcinoma (HCC) has become one of the most common leading causes of cancer-related deaths worldwide^1^. Despite advancement in the treatment modalities for HCC such as surgery, liver transplantation, chemotherapy, and radiotherapy, the 5-year overall survival rate of HCC patients has hardly improved^2,3^. The high mortality and poor prognosis of HCC are attributed to the incomplete understanding of the molecular mechanisms underlying the development and progression of HCC. In this regard, further understanding the molecular mechanisms is of great clinical significance for developing novel therapeutic targets for HCC treatment.

Recently, numerous researches have been focused on the long non-coding RNAs (lncRNAs), which are non-protein transcripts with the length more than 200 nt^4^. The lncRNAs are found to involve in a diverse biological processes including transcriptional regulation, splicing, chromatin modification *et al*.^5,6^. LncRNAs have been suggested to serve as diagnostic and prognostic biomarkers because studies have found that dysregulation of lcnRNAs were associated with cancer development in various types of cancers. LncRNAs such as TUG1, Linc00152 and CCAT1 were found to involve in the pathogenesis in colorectal cancer^7–9^. Further,XIST, GAS5-AS1, HOTAIR and MALAT1 are found to play key roles in the development and progression of lung cancer^10–13^. In HCC, an increasing number of lncRNAs have been reported to be associated with cancer development. PVT1 has been found to serve as a biomarker for HCC prognosis^14^. HULC promotes tumorigenesis and metastasis via epithelial-mesenchymal transition in HCC^15^. HOTAIR was found to promote human liver cancer stem cell growth through down-regulation of SETD2^16^. The expression of lncRNA, SPRY4 intronic transcript 1 (SPRY4-IT1), transcribed from an intron, is found to be up-regulated in various types of cancers including colorectal cancer, esophageal squamous cell carcinoma, glioma, and bladder cancer^17–20^. However, the role of SPRY4-IT1 in other types of cancers remains unknown, particularly in HCC.

The estrogen-related receptor α (ERRα) belongs to the nuclear receptor family. Because there is no natural ligand of ERRα has been identified to date, ERRα is regarded as an “orphan” receptor. It regulates the target genes in a ligand-independent way^21^. ERRα is found to involve in various physiological processes such as innate immunity, energy metabolism and osteoblast differentiation as well as bone formation^22–25^. Recently, ERRα has been suggested to be associated with the development and progression of various types of cancers. Knock-down of ERRα inhibited the tumor development on uterine endometrial cancer;^26^ ERRα is a marker of tamoxifen response and survival in triple-negative breast cancer;^27^ ERRα was also found to coordinate colon cancer cell proliferation and tumorigenic capacity with energy metabolism;^28^ WNT11 expression is induced by ERRα and beta-catenin, and acts in an autocrine manner to increase cancer cell migration^29^. However, whether ERRα plays a role in HCC development is still unclear.

In this study, we identified the up-regulation of SPRY4-IT1 in HCC tissues, which was associated with poor prognosis in HCC patients. We also found that high level of SPRY4-IT1 promoted proliferation, cell cycle progression and suppressed cell apoptosis of HCC. Furthermore, SPRY4-IT1 was found to function in the progression of HCC via interacting with ERRα.

## Materials and Methods

### Human tissues samples

A total of 82 patients with HCC were included in this study. All the patients had undergone routine hepatic resection at the Fifth Affiliated hospital of Guangzhou Medical University from 2012 to 2015, and none of the patients had received chemotherapy or radiotherapy prior to surgical resection. This study was approved by the Ethics Committee of the Fifth Affiliated hospital of Guangzhou Medical University. All patients provided written informed consent for the use of tissue samples for clinical research. The histological diagnosis and differentiation of tumors were evaluated by pathologists according to the criteria of WHO classification system. The clinicopathological features are shown in Table 1. All the tissue samples were snap-frozen in liquid nitrogen immediately following surgical resection, and stored in −80 °C for further experimentation.

**Table 1:** Correlation between SPRY4-IT1 expression levels and clinicopathological characteristics in HCC patients.

Characteristics	Number of patients	Low	High	*P* value
Age (years)				0.822
<55	30	13	17	
≥55	52	24	28	
Gender				0.805
Male	60	26	32	
Female	22	9	13	
Serum AFP (ng/ml)				0.246
<20	29	16	13	
≥20	53	21	32	
HBsAg				0.685
Negative	6	2	4	
Positive	76	35	41	
Tumor size (cm)				0.118
<5	32	18	14	
≥5	50	19	31	
Liver cirrhosis				0.179
Absence	19	11	7	
Presence	63	26	38	
Histological differentiation				0.1737
Well	12	7	5	
Moderate	32	17	15	
Poor	38	13	25	
TNM stage				0.03
I + II	34	22	12	
III + IV	48	15	33	
Metastasis				0.002
No	47	28	19	
Yes	35	9	26	

Low, low expression level of SPRY4-IT1; High, high expression level of SPRY4-IT1.

### Cell culture

The normal liver cell line, HL7702 and the human liver cancer cell lines MHCC97L, MHCC97H, HepG2 and SMMC7721 were obtained from the Chinese Academy of Sciences (Shanghai, China). All the cells were cultured in Dulbecco’s modified Eagle’s medium (DMEM) with 10% fetal bovine serum (FBS, Thermo Scientific, Waltham, USA) in a humidified atmosphere at 37 °C with 5% CO_2_.

### SiRNAs and vector construction

All the siRNAs (SPRY4-IT1 siRNA: CCCAGAATGTTGACAGCTGCCTCTT or its respective scrambled siRNA; ERRα siRNA or its respective scrambled siRNA) were purchased from Genechem (Shanghai, China). The ERRα overexpression constructs or the empty vector were purchased from Genepharma (Shanghai, China). All the transfections were performed by using the Lipofetamine reagent (Invitrogen) according to the manufacturer’s instructions.

### Quantitative real-time PCR (qRT-PCR)

Total RNA from tissue samples or cells were extracted using Trizol reagent (Invitrogen, Carlsbad, USA), and the RNA was reversely transcribed to cDNA by using PrimeScript RT-polymerase (Takara, Dalian, China). Real-time PCR was performed using the Applied Biosystems 700 Sequence Detection System (Applied Biosystems, Foster City, USA). GAPDH was used as an internal control. The relative expressions of SPRY4-IT1 and ERRα were normalized to GAPDH expression levels by using the 2-∆∆Ct method. The primers for SPRY4-IT1 are: forward, 5′-AGCCACATAAATTCAGCAGA-3′, reverse, 5′-CGATGTAGTAGGATTCCTTT-3′; the primers for ERRα are: forward, 5′-CAGGAAAGTGAATCCCAG-3′, reverse, 5′-CTTTGCAGCAAATATACATT-3′; the primers for GAPDH are: forward, 5′-GTCGGAGTCAACGGATTTGG-3′ reverse, 5′-AAAAGCAGCCCTGGTGACC-3′.

### Western blotting assay

The HepG2 and SMMC7721 cells were lysed with denaturing SDS-PAGE sample buffer using standard methods. Protein lysates were separated on a 10% SDS-PAGE gel and transferred to the nitrocellulose membranes. The membranes were blocked with 5% skimmed non-fat milk for 1 h at room temperature, and then the membrane were incubated with rabbit polyclonal anti-ERRα antibody (Abcam, Cambridge, USA) at 4 °C overnight. After primary antibody incubation, the membranes were then incubated with HRP-conjugated anti-IgG at room temperature for 2 h. Signal was detected by an ECL system (Amersham Pharmacia, Piscataway, USA).

### CCK-8 assay

Twenty-four hours after transfected with SPTRY4-IT1 siRNA, or its respective scrambled siRNA; ERRα siRNA or its respective scrambled siRNA; or after co-transfected with scrambled siRNA+ empty vector, SPRY4-IT1 siRNA+ empty vector, or SPRY4-IT1 siRNA + ERRα overexpressing vector, cells were seeded in the 96-well plate at a concentration of 5 × 10^3^ cells, and further cultured for 24 h, 48 h and 72 h accordingly. Cell viability was determined by a Cell Counting Kit-8 (Beyotime, Shanghhai, China) according to the manufacturer’s instructions. The cell viability index was detected at the wavelength of 450 nm using the Elx800 reader (Bio-Tek Instruments, Winooski, USA).

### Colony formation assay

Twenty-four hours after transfection (see CCK-8 assay section), cells were seeded in six-well plates containing DMEM medium supplemented with 10% FBS. After 24 h, the medium was replaced with new medium containing G418. After further culturing for 14 days, cells were fixed with methanol and stained with 0.1% crystal violet. Visible colonies were manually counted.

### Invasion and migration assays

Cell invasion and migration was evaluated using Transwell assay. For cell migration assay, twenty-four hours after transfection (see CCK-8 assay section), cells at a concentration of 5 × 10^5^ were seeded in the top chamber with the non-coated membrane transwell (8 μm pore size inserts, BD Biosciences, San Jose, USA). For the cell invasion assay, matrigel (BD Biosciences) was polymerized in the transwell inserts for 45 min at 37 °C. For both assays, cells were plated in the top chamber in medium without FBS, while the lower chamber was filled with 10% FBS and EGF (Sigma, St Louis, USA). Cells were further incubated for 24 h and the cells that did not migrate or invade through the pores were removed by a cotton swab. Cells on the lower surface of the membrane were stained with crystal violet and counted under a microscope.

### Flow cytometry

Twenty-four hours after transfected with SPTRY4-IT1 siRNA, cells were harvested by trypsinization. After the double staining with FITC-Annexin V and propidium iodide, cell apoptosis detection was performed by using the FITC Annexin V Apoptosis Detection Kit (BD Biosciences) according to the manufacturer’s instructions. The cell apoptosis was analyzed with a flow cytometer (FACScan; BD Biosciences) equipped with a Cell Quest Software (BD Biosciences). For the cell cycle analysis, cells were stained with propidium oxide by using the Cycle TEST PLUS DNA Reagent Kit (BD Biosciences) following the manufacturer’s instructions and analyzed by FACScan.

### Statistical analysis

GraphPad Prism software was used for data analysis. All the data are shown as mean ± SD, and data were analyzed by t-test (for comparing two groups) or by one-way ANOVA followed by Turkey’s multiple comparison tests (for comparing more than two groups). Results were considered statistically significant at *P* < 0.05.

## Results

### SPRY4-IT1 mRNA is up-regulated in HCC tissues and is correlated with poor prognosis in HCC patients

**Figure 1:**
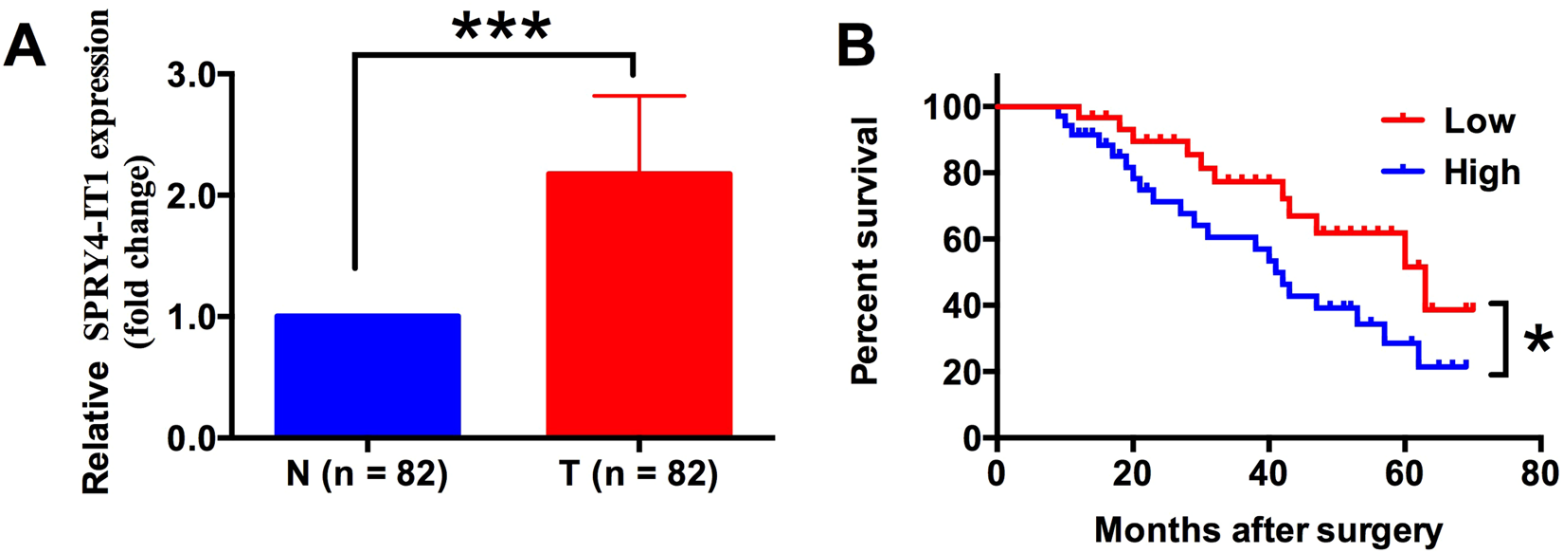
Relative SPRY4-IT1 RNA expression and its association with overall survival of HCC patients. (**A**) Relative SPRY4-IT1 RNA expression was examined by qRT-PCR in 82 pairs of cancerous liver tissues and adjacent non-cancerous liver tissues from HCC patients, ***P < 0.001 (paired t-test). (**B**) Kaplan-Meier survival curve. Patients were divided into SPRY4-IT1-low (Low) and SPRY4-IT1-high (High) groups based on the median of relative SPRY4-IT1 RNA expression in HCC. *P < 0.05.

We evaluated the RNA levels of SPRY4-IT1 in 82 pairs of human primary HCC tissues and the respective adjacent normal non-cancerous liver tissues using qRT-PCR. We found that the expression of SPRY4-IT1 in HCC tissues were significantly higher than that examined in the respective adjacent normal non-cancerous liver tissues (Fig. 1A, *P* < 0.001). Clinical analysis demonstrated that SPRY4-IT1 RNA levels were correlated with TNM stage and metastasis (Table 1, *P* < 0.05). However, we have not observed significant correlation between SPRY4-IT1 expression levels and other clinical characteristics such as age, gender, serum AFP level, HBsAg status, tumor size, liver cirrhosis and histological differentiation (Table 1). To understand the prognostic role of SPRY4-IT1 in HCC, we examined the correlation between SPRY4-IT1 expression and HCC patients’ overall survival. The results showed that high level of SPRY4-IT1 was significantly correlated with poor 5-year overall survival rate in HCC patients (Fig. 1B, *P* < 0.05).

### SPRY4-IT1 knock-down inhibits cell proliferation, colony formation, cell invasion and migration in HCC cell lines

**Figure 2:**
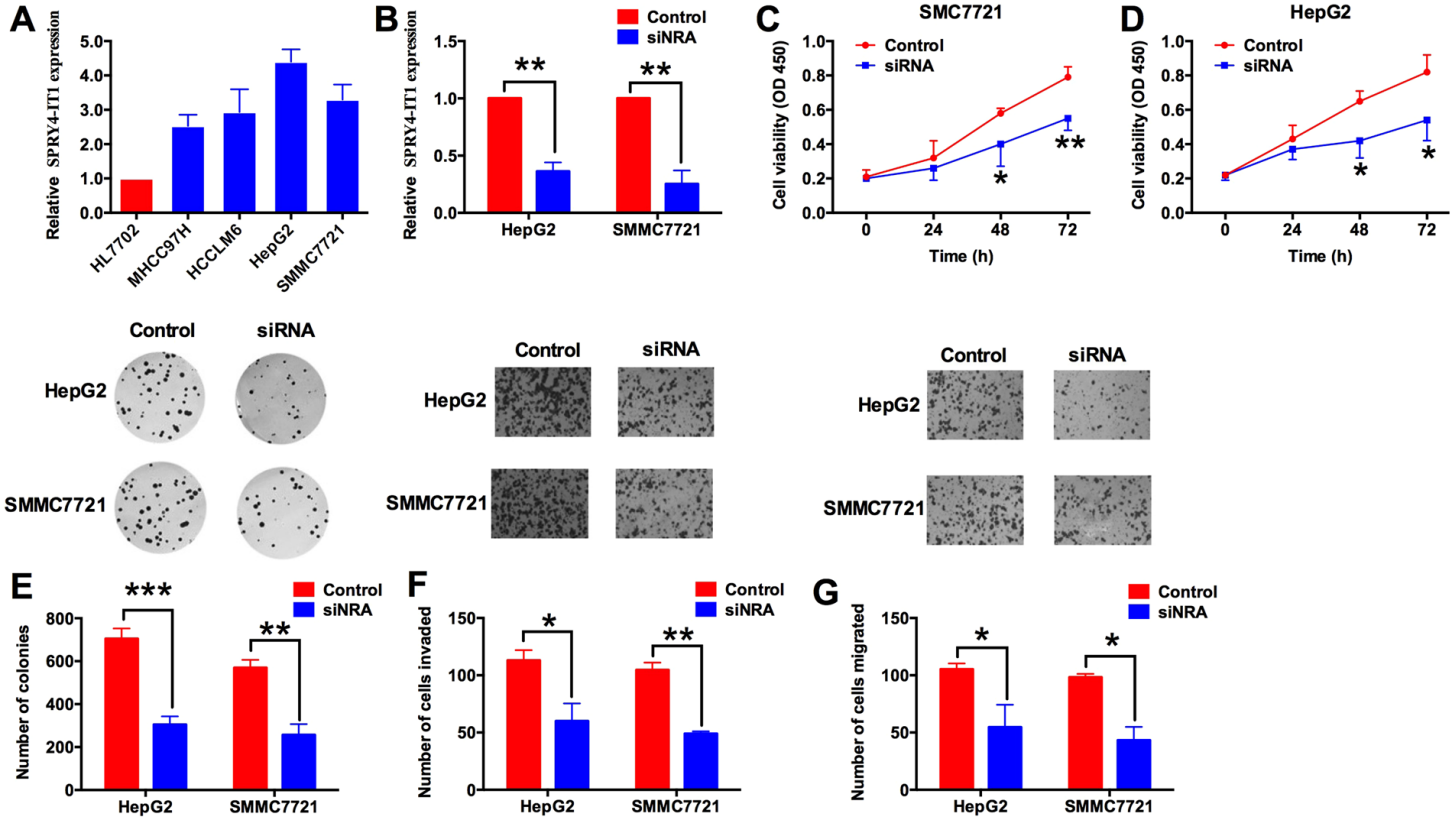
Knock-down of SPRY4-IT1 suppresses cell proliferation, colony formation, cell invasion and migration in HCC cells. (**A**) Relative SPRY4-IT1 expression levels in different liver cell lines measured by qRT-PCR. (**B**) Relative SPRY4-IT1 expression levels in scrambled siRNA (control) or SPRY4-IT1 siRNA (siRNA) transfected HepG2 and SMMC2117 cells were examined by qRT-PCR. (**C**) and (**D**) Cell viability in scrambled siRNA (control) or SPRY4-IT1 siRNA (siRNA) transfected HepG2 and SMMC2117 cells determined by CCK-8 assay at 0 h, 24 h, 48 h and 72 h time point. (**E**) Colony formation assay performed in HepG2 and SMMC2117 cells transfected with siRNA (control) or SPRY4-IT1 siRNA (siRNA). (**F**) Cell invasion and (**G**) migration assays performed in scrambled siRNA (control) or SPRY4-IT1 siRNA (siRNA) transfected HepG2 and SMMC2117 cells. Data are shown as mean ± SD, significant differences were marked as *P < 0.05, **P < 0.01, ***P < 0.001.

The expression of SPRY4-IT1 was further examined *in vitro* in one normal liver cell line (HL7702) and four HCC cell lines (MHCC97H, HCCLM6, HepG2 and SMMC7721) by qRT-PCR. We found that the relative expression of SPRY4-IT1 in HCC cell lines were higher than that in normal liver cell line, with the highest expression in HepG2 and second highest in SMMC7721 cell lines (Fig. 2A, P < 0.05). In the following study, HepG2 and SMMC7721 cell lines were chosen for further functional investigation *in vitro*. We transfected the siRNA targeting SPRY4-IT1 to knock-down SPRY4-IT1 in HepG2 and SMMC7721 cell lines. The SPRY4-IT1 siRNA transfection in HepG2 and SMMC7721 cells significantly reduced the expression levels of SPRY4-IT1 (Fig. 2B, P < 0.05). To examine the role of SPRY1-IT1 knock-down in cell proliferation, CCK-8 assay was performed at 0 h, 24 h, 48 h and 72 h after SPRY1-IT1 transfection. In comparison with scrambled siRNA (Control) transfected HepG2 and SMMC7721 cells at 48 or 72 h, HepG2 and SMMC7721 cells transfected with SPRY4-IT1 siRNA had significantly lower proliferative ability (Fig. 2C and D, *P* < 0.05). To further investigate the effect of SPRY4-IT1 knock-down on cell growth, we performed colony formation assay. The results showed that the number of colonies in SPRY4-IT1 transfected HepG2 and SMMC7721 cells were significantly lower than that in scrambled siRNA transfected cells (Fig. 2E, P < 0.05). To examine the effect of SPRY4-IT1 knock-down on cell invasive and migratory abilities, we performed the cell invasion and cell migration assays. As shown in Fig. 2F and G, the number of invaded and migrated cells in SPRY4-IT1 transfected HepG2 and SMMC7721 cells were significantly reduced when compared to scrambled siRNA transfected cells (Fig. 2F and G, P < 0.05).

### SPRY4-IT1 knock-down induces cell cycle arrest and cell apoptosis

**Figure 3:**
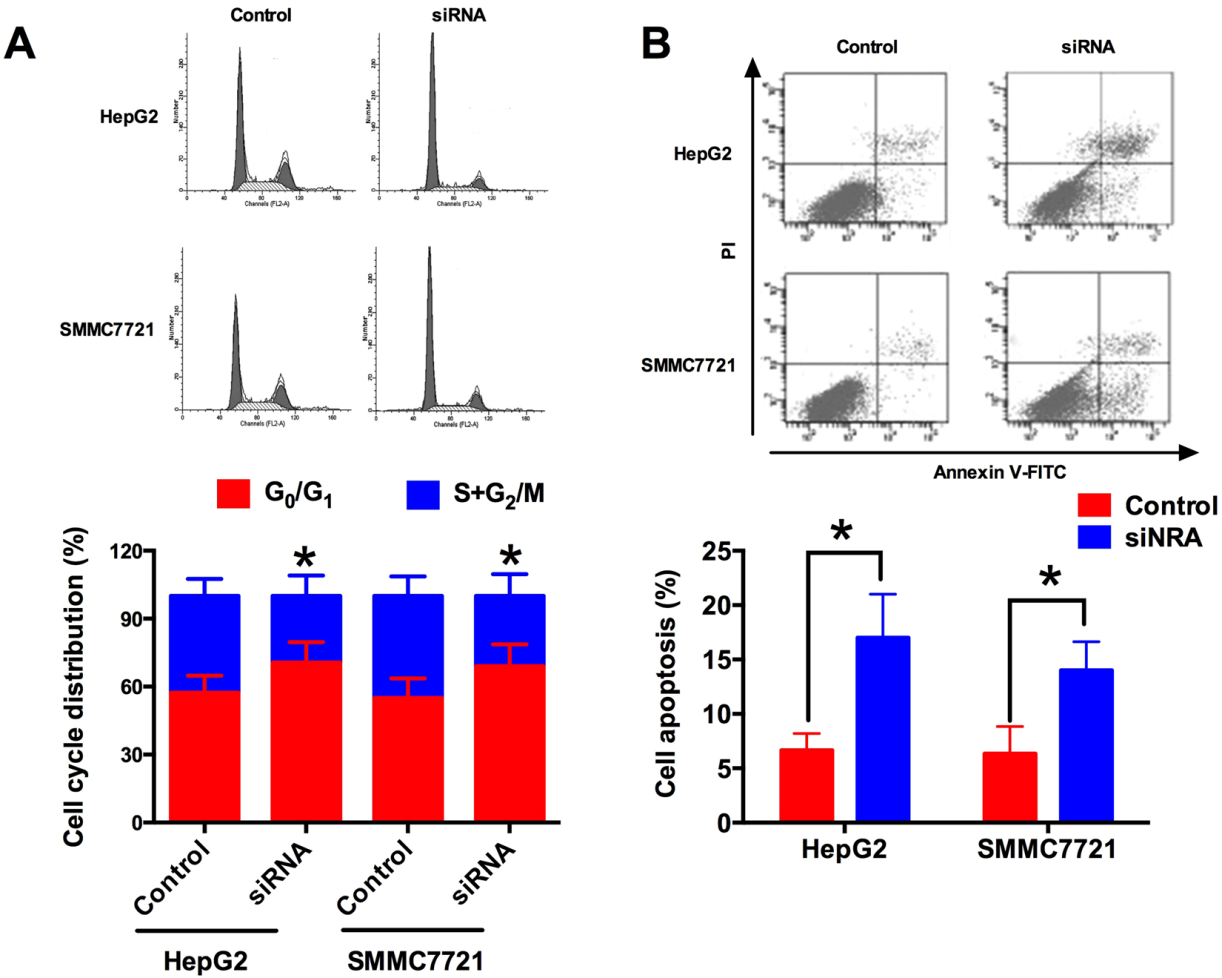
Know-down of SPRY4-IT1 induces cell cycle arrest and apoptosis. (**A**) Cell cycle prolife in scrambled siRNA (control) or SPRY4-IT1 siRNA (siRNA) transfected HepG2 and SMMC2117 cells examined by flow cytometry with propidium iodide staining. (**B**) Cell apoptosis in scrambled siRNA (control) or SPRY4-IT1 siRNA (siRNA) transfected HepG2 and SMMC2117 cells examined by flow cytometry. Data are shown as mean ± SD, significant differences were marked as *P < 0.05.

The cell cycle and cell apoptosis in HCC cell lines were analyzed by flow cytometry. Compared with the scrambled siRNA transfected HepG2 and SMMC7721 cells, SPRY4-IT1 transfected cell lines had higher proportion of cell population at G0/G1 phase with lower proportion of cell population at S + G2/M phases (Fig. 3A, P < 0.05). In addition, SPRY4-IT1 transfection in HepG2 and SMMC7721 cells significantly increased the cell apoptotic rate when compared with scrambled siRNA transfection in these cell lines (Fig. 3B, P < 0.05).

### SPRY4-IT1 knock-down suppresses ERRα expression, and ERRα knock-down suppressed cell proliferation, colony formation, cell invasion and cell migration

**Figure 4:**
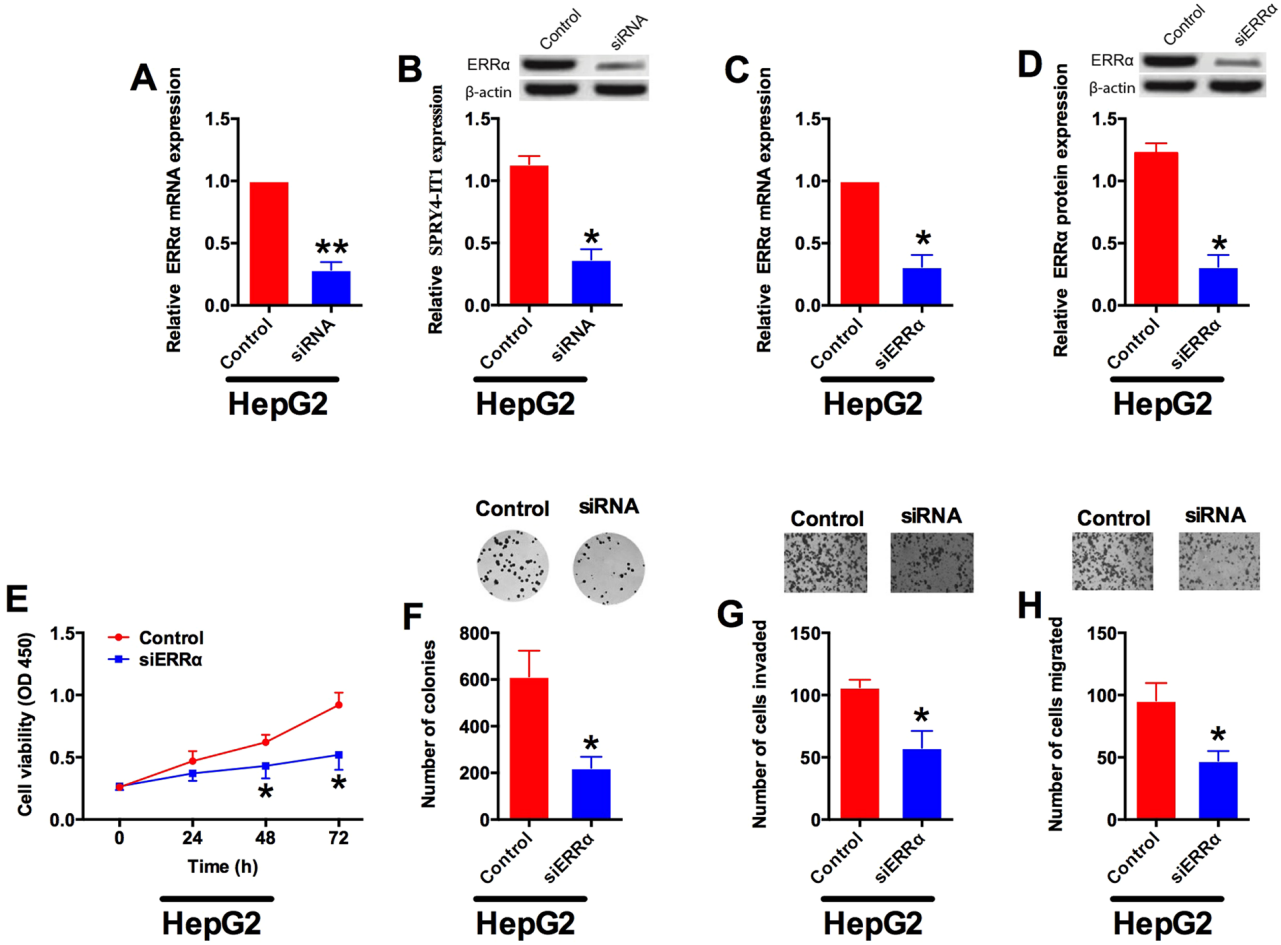
Knock-down of ERRα suppresses cell proliferation, colony formation, cell invasion and migration in HCC cells. (**A**) The mRNA and (**B**) protein expression of EERα in scrambled siRNA (control) or SPRY4-IT1 siRNA (siRNA) transfected HepG2 cells examined by qRT-PCR and Western blotting. (**C**) The mRNA and (**D**) protein expression of EERα in scrambled siRNA (control) or EERα siRNA (siRNA) transfected HepG2 cells examined by qRT-PCR and Western blotting. (**E**) Cell viability in scrambled siRNA (control) or EERα siRNA (siRNA) transfected HepG2 cells determined by CCK-8 assay at 48 h time point. (**F**) Colony formation assay performed in scrambled siRNA (control) or EERα siRNA (siRNA) transfected HepG2 cells. (**G**) Cell invasion and (**H**) migration assays performed in scrambled siRNA (control) or EERα siRNA (siRNA) transfected HepG2 cells. Data are shown as mean ± SD, significant differences were marked as *P < 0.05, **P < 0.01.

To investigate if SPRY4-IT1 had an interaction with ERRα, we performed qRT-PCR and Western blotting to examine the RNA and protein expression of ERRα in SPRY4-IT1 siRNA or scrambled siRNA transfected HCC cell line. As shown in Fig. 4A and B, SPRY4-IT1 siRNA transfection HepG2 cells significantly suppressed the mRNA and protein expression levels of ERRα (Fig. 4A and B). Furthermore, we examined the role of ERRα in HCC cell proliferation, colony formation, cell invasion and migration. ERRα inhibitory transfection significantly suppressed the mRNA and protein expression of ERRα in HepG2 cells (Fig. 4C and D, P < 0.05). The CCK-8 results showed that, HepG2 cells transfected with ERRα siRNA had significantly suppressed proliferating ability in comparison with scrambled siRNA (Control) transfected HepG2 cells at 48 h (Fig. 4E, *P* < 0.05). To further investigate the effect of ERRα knock-down on cell growth, colony formation assay was also performed. The results showed that the number of colonies in ERRα siRNA transfected HepG2 cells were reduced compared with scrambled siRNA transfected cells (Fig. 4F, P < 0.05). To examine the effect of ERRα knock-down on cell invasive and migratory abilities, cell invasion assay and cell migration assay were performed, and the results demonstrated that the number of invaded and migrated cells in ERRα transfected HepG2 cells were significantly lower than that in scrambled siRNA transfected cells (Fig. 4G and H, P < 0.05).

### ERRα overexpression antagonized the effects of SPRY4-IT1 knock-down on cell proliferation, colony formation, cell invasion and migration in HCC cell lines

**Figure 5:**
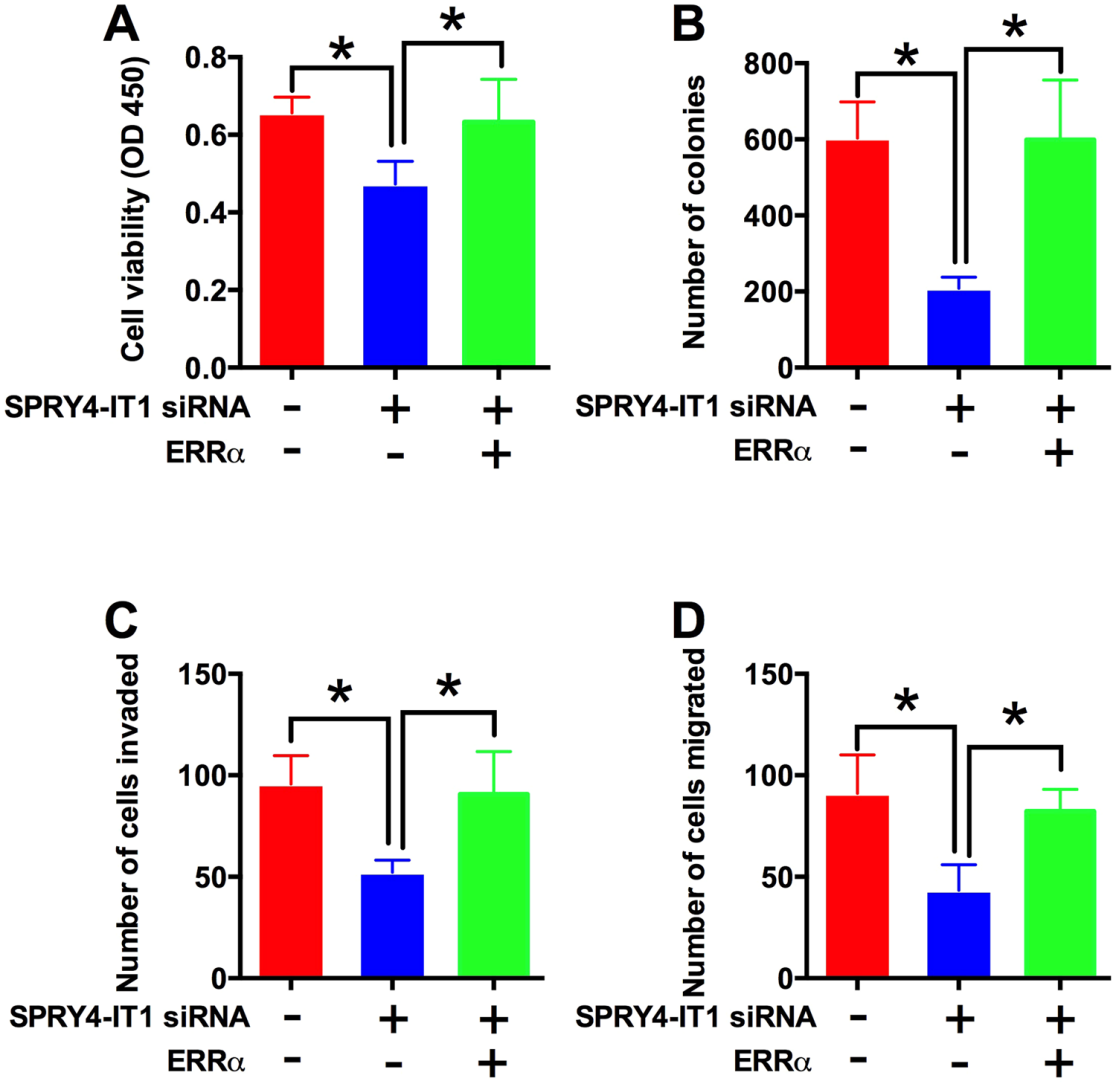
ERRα overexpression antagonized the effects of SPRY4-IT1 knock-down on cell proliferation, colony formation, cell invasion and migration in HCC cells. (**A**) Cell viability in scrambled siRNA+ empty vector, SPRY4-IT1 siRNA+ empty vector, or SPRY4-IT1 siRNA+ ERRα overexpressing vector transfected HepG2 cells determined by CCK-8 assay at 48 h time point. (**B**) Colony formation assay performed in scrambled siRNA+ empty vector, SPRY4-IT1 siRNA+ empty vector, or SPRY4-IT1 siRNA+ ERRα overexpressing vector transfected HepG2 cells determined by CCK-8 assay at 48 h time point. (**C**) Cell invasion and (**D**) migration assays performed in scrambled siRNA+ empty vector, SPRY4-IT1 siRNA+ empty vector, or SPRY4-IT1 siRNA+ ERRα overexpressing vector transfected HepG2 cells. Data are shown as mean ± SD, significant differences were marked as *P < 0.05.

To further confirm the interaction between SPRY4-IT1 and ERRα, HepG2 cells were transfected with scrambled siRNA+ empty vector, SPRY4-IT1 siRNA+ empty vector, or SPRY4-IT1 siRNA+ ERRα overexpressing vector. After transfection, CCK-8, colony formation, cell invasion and migration assays were performed. In consistent with previous results, co-transfection with SPRY4-IT1 siRNA and empty vector in HepG2 cells significantly suppressed cell proliferation, colony formation, cell invasion and migration in comparison with co-transfection with scrambled siRNA+ empty vector (Fig. 5, P < 0.05). Further, co-transfection with SPRY4-IT1 siRNA+ ERRα overexpressing vector in HepG2 cells increased the cell proliferation, colony formation, cell invasion and migration in comparison with co-transfection with SPRY4-IT1 siRNA + empty vector (Fig. 5, P < 0.05).

## Discussion

LncRNAs have become increasingly recognized as important regulators of gene expressions, as lncRNAs can regulate protein-coding genes at epigenetic, transcriptional, and post-transcription levels^5^. Recently studies have shown that a lot of lncRNAs are frequently aberrantly expressed in various types of cancers, and the dysregulation of lncRNAs have been suggested to be associated with tumor pathogenesis and metastasis. They are also useful in tumor diagnosis and prognosis^6^. Therefore, it is necessary to understand the molecular mechanisms of lncRNAs in cancer development and progression.

In this study, we detected the up-regulation of SPRY4-IT1 RNA in the HCC tissues when compared to adjacent non-cancerous liver tissues. Furthermore, the expression of SPRY4-IT1 was significantly correlated with TNM stage and tumor metastatic status. This was consistent with a previous study which also showed that SPRY4-IT1 was up-regulated in HCC tissues and was correlated with tumor differentiation, tumor size, and TNM stage^30^. Furthermore, we performed the loss-of-function experiments to look into the *in vitro* molecular mechanisms of SPRY4-IT1 in HCC development. The *in vitro* results showed that knock-down of SPRY4-IT1 suppressed the cell proliferation, colony formation, cell invasion and migration in HCC cell lines, and the results were in agreement with previous studies showing that knock-down of SPRY4-IT1 suppressed the cell proliferation, colony formation, cell invasion and migration in several types of cancers including colorectal cancer, esophageal squamous cell carcinoma, prostate cancer, glioma, gastric cancer, bladder cancer and breast cancer^17–20^. Thus, our results may suggest the oncogenic role of SPRY4-IT1 in the pathogenesis of HCC. In addition, the flow cytometry was performed to investigate the mechanistic role of SPRY4-IT4 in cell cycle and cell apoptosis. We found that knock-down of SPRY4-IT1 induced G_0_/G_1_ cell cycle arrest and also increased the apoptotic rate of HCC cell lines. Similarly, in other types of cancers such as esophageal squamous cell carcinoma, breast cancer, lung cancer, melanoma, knock-down of SPRY4-IT1 also induced G_0_/G_1_ cell cycle arrest and cell apoptosis^18,31–33^. Therefore, these results indicate that knock-down of SPRY4-IT1 inhibited HCC progression by inducing cell cycle arrest as well as cell apoptosis.

The roles of ERRα in cancer development have been revealed in recent studies. In breast cancer, ERRα has been extensively studied, in which dysregulation of ERRα not only contributes to the progression of breast cancer, but also closely associated with the chemo-resistance of breast cancers^34^. In addition, ERRα was also found to play an important role in prostate cancer. ERRα augments HIF-1 signaling by directly interacting with HIF-1α in normoxic and hypoxic prostate cancer cells^35^. However, the role of ERRα in HCC is still unclear. The elevated levels of ERRα are associated with the increased cell proliferation and migration in breast cancer and prostate cancer cells^36,37^. In the present study, we demonstrated that knock-down of EERα significantly inhibited cell proliferation, colony formation, cell invasion and migration. Moreover, we examined whether SPRY4-IT1 had an interaction with ERRα, and the transfection study showed that SPRY4-IT1 knock-down also suppressed the expression of ERRα in HCC cells. More importantly, ERRα overexpression antagonized the effects of SPRY4-IT1 knock-down on HCC cell progression. These results may suggest that SPRY4-IT1 regulated HCC progression via interacting with ERRα. However, our results only showed the preliminary findings about the interaction between SPRY4-IT1 and ERRα. In order to find out more detailed molecular mechanisms underlying these interactions, more experiments should be performed in the future studies.

In conclusion, the present study showed that SPRY4-IT1 was up-regulated in HCC tissues and was associated with poor prognosis in HCC patients. Knock-down of SPRY4-IT1 inhibited cell proliferation, colony formation, cell invasion and migration at least via interacting with ERRα. These results suggest that SPRY4-IT1 may serve as a novel target for the management of HCC or as a biomarker for HCC diagnosis or prognosis, though the detailed molecular mechanisms of SPRY4-IT1 on HCC development may require further investigation.
